# Psychological Consequences Associated With Coronary Artery Bypass Graft Surgery: A Bibliometric Analysis

**DOI:** 10.7759/cureus.29331

**Published:** 2022-09-19

**Authors:** Moli Jain, Vishnu Vardhan, Pallavi Harjpal

**Affiliations:** 1 Physiotherapy, Ravi Nair Physiotherapy College, Datta Meghe Institute of Medical Sciences, Wardha, IND; 2 Cardiorespiratory Physiotherapy, Ravi Nair Physiotherapy College, Datta Meghe Institute of Medical Sciences, Wardha, IND

**Keywords:** vascular psychiatry, psychological factors, anxiety, depression, coronary artery bypass graft, bibliometric analysis

## Abstract

The current paper explores the mutual impact of psychological factors and cardiac surgery on each other using bibliometric analysis with the help of indicative cited publications, co-cited journals, and collaborations between countries, institutions, and authors. Citation analysis is an attractive methodology because it provides quantitative information that is readily acquired with electronic databases and that can be compared across topic areas. The bibliometric investigation was done using the PubMed database, Scopus, Web of Science, etc. with the help of appropriate MeSH words. Followed by an analysis of data with the help of CiteSpace 5.3.R4, Microsoft Excel 2016, and IBM SPSS Statistics 20.0 software (IBM Corp., Armonk, NY). The software extracts the number of scientific publications, citation frequency, and keyword trends. Relational figures and tables were obtained for data interpretation. The records of 259 articles were analyzed using bibliometric investigation which shows the increasing incidence of psychological consequences linked with coronary artery bypass grafting (CABG) surgery. This indicates that immediate management is required to overcome this. The present bibliometric study emphasizes the need for psychological screening and management of post-CABG patients. This will lay the path for organizing and prioritizing future research on vascular psychiatry and its early management.

## Introduction and background

Coronary artery disease (CAD) is one of the foremost causes of fatality worldwide. To decrease suffering and increase care, rapid advances are being undertaken leading with coronary artery bypass grafting (CABG) [1]. It is a revascularization surgery commonly performed to redirect blood to the myocardium. Anchored to this, a strong biological link is well established between emotional and psychological factors and CAD [2,3]. Studies have shown that psychological consequences are associated not only with CAD but also with individuals who have undergone CABG surgery. This association has been found to have a direct link in the short and long term with increased chances of rehospitalization, delay in recovery, and early mortality [4]. This creates room to incorporate psychiatric screening and management for individuals with diseases associated with blood vessels. Hence, vascular psychiatry emerged as a new concept highlighting the need for psychiatric intervention for vascular syndromes such as vascular depression, vascular cognitive impairment, anxiety, and depression following cardiac disease and its long-term conservative and surgical management [5].

The current paper explores the mutual impact of psychological factors and cardiac surgery on each other. The emergence of emotions may be due to sudden deterioration in health hampering daily activities, lack of awareness about the disease, and fear of death [6]. This leads to a vascular syndrome that can develop immediately after the acute disease stage, due to recurrent hospitalization, during long waiting for the surgery, and even after post-surgery [7,8]. Persistence of these factors postoperatively and even after discharge for two or more weeks may invite other consequences which require immediate attention [9].

Depression has been the leading independent prognostic factor associated with CABG in approximately one-third of patients followed by anxiety and stress [10,11]. In such patients, the presence and persistence of depressive disorder and anxiety become a barrier to participating in a cardiac rehabilitation program which further compromises recovery and increases healthcare issues and costs. Such observations open a window of opportunity for anchoring psychosocial intervention along with cardiac rehabilitation to gain better outcomes among such patients [12].

Bibliometric analysis is a qualitative statistical tool used by an investigator to gain in-depth knowledge about the scientific impact of a study, citation count, institutions, authors, collaborations worldwide, and keyword trends in the research field of a study. Citation analysis is an attractive methodology because it provides quantitative information that is readily acquired with electronic databases and that can be compared across topic areas [13]. To better understand the psychological consequences associated with CABG, bibliometric citation analysis can be done. Thus, the current study aimed to conduct a bibliometric analysis to summarize the psychological factors associated with CABG surgery with the help of indicative cited publications, co-cited journals, and collaborations between countries, institutions, and authors.

## Review

Methodology

Data Source

The initial step in any bibliometric investigation is to choose the right database to hunt for the appropriate paperwork. PubMed databases, Scopus, Web of Science, and Cochrane Library were searched for articles published between the years 1994 to 2020.

Search Strategy

In any bibliometric investigation, the second difficulty is to create a legitimate search query that returns as many papers as feasible while minimizing irrelevant (false-positive) results. In the present study, the authors establish a comprehensive search query to gather all potential research focusing on CABG surgery and its psychological consequences. The search keywords were established using Medical Subject Heading (MeSH) terms from PubMed. The search strategy was as follows: Title = (Coronary artery bypass graft OR Coronary artery bypass graft surgery OR CABG*) AND Title = (Depression and Anxiety OR Depression OR Anxiety OR Post-operative depression) AND Language=English.

Analysis Tool

The records of 259 articles were analyzed using CiteSpace 5.3.R4, Microsoft Excel 2016, and IBM SPSS Statistics 20.0 software (IBM Corp., Armonk, NY) specifically designed for data analysis. The java-based CiteSpace 5.3.R4 was frequently used to display and analyze networks. Data extracted from the PubMed database was in tabular form. Microsoft Excel 2016 was used to tabulate data and construct a trending figure of publication quantity with years. In addition, SPSS Statistics 20.0 software was used to carry out Pearson’s correlation analysis of year and publication quantity.

Data Extraction

After devising the search strategy, two authors (MJ and VV) extracted the literature and bibliometric indicators, respectively. An appropriate analysis tool was used to extract the number of scientific publications, citation frequency, and keyword trends. Relational figures and tables were obtained, for data interpretation. First, the examination of annual scientific production and collaboration between nations, institutions, and authors. Second, study the geographical distribution and trends of journals, countries, institutions, and authors over years. And the last, the Analysis of keywords and their growth trend over years are the following aspects used for global trends and interpretation of scientific research findings on psychological factors linked with CABG surgery.

Result

Analysis of Document

**Figure 1:**
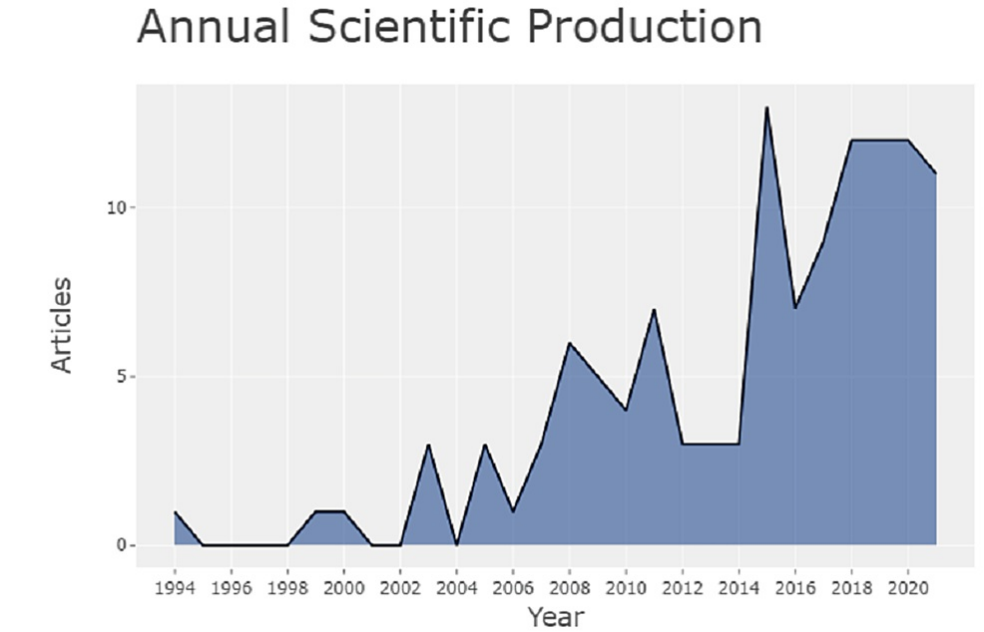
A graphical illustration of annual scientific production between the years 1994 and 2020.

The record extracted from the PubMed database about psychological consequences associated with Coronary artery bypass graft surgery from the year 1994 to the year 2020 generated a list of 120 documents from 98 sources. The annual scientific production as shown in Figure 1 was highest in the year 2015 with 13 publications, followed by a steady rate of 12 publications per year from 2018 to 2020. The progress of the annual growth of research articles is seen after the year 2015. This indicates growing attention toward psychological factors associated with CABG surgery annually.

Analysis of top authors and their production over time

**Figure 2:**
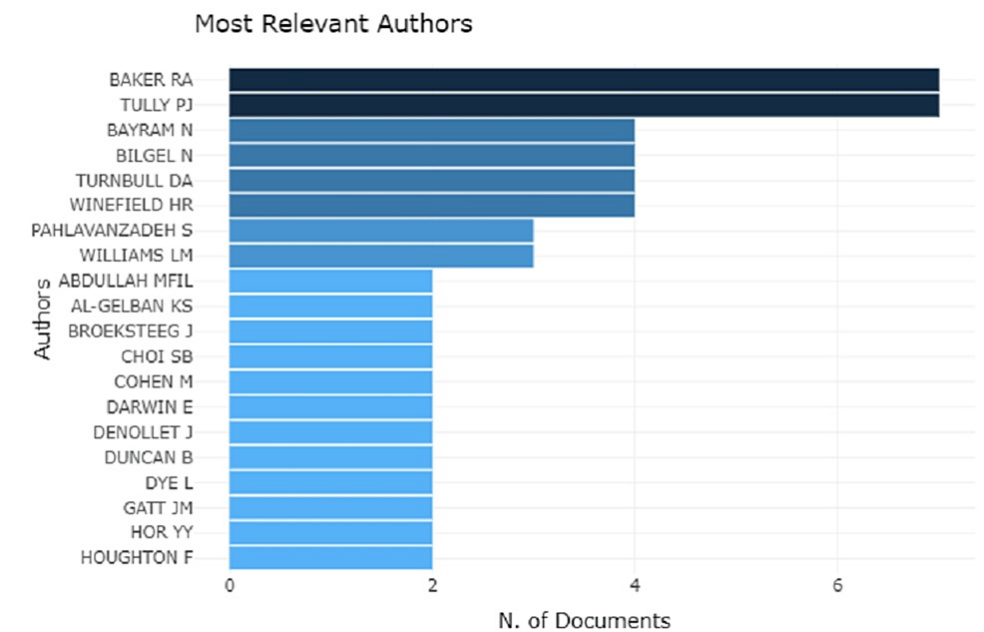
A graphical illustration of the analysis of top authors with their number of documents.

**Figure 3:**
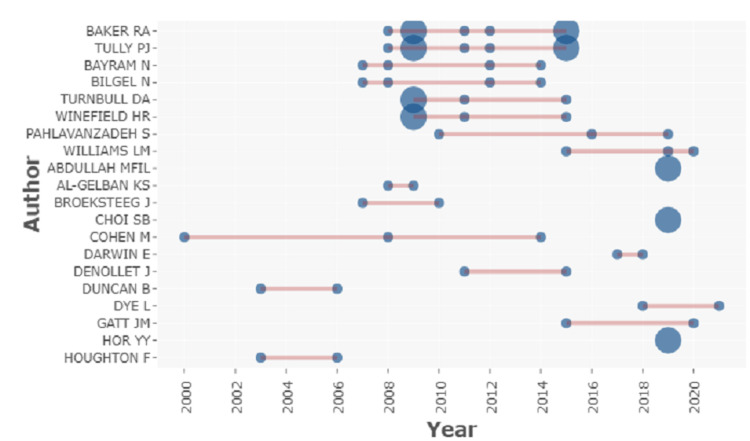
A graphical illustration of the analysis of the top author’s annual production over time.

A total of 551 authors list was generated. The interpretation based on top authors and their production over time shows Cohen has the highest production span from the year 2000 to 2014 with three articles. On the other hand, Baker et al. and Tully et al. are at the peak with seven articles (Figure 2) over a time span between the years 2008 and 2015 (Figure 3). Following this, Bilgen et al. and Bayram et al. had four articles (Figure 2) between the years 2007 and 2014 (Figure 3).

Analysis of Journal and its Growth Trends

**Figure 4:**
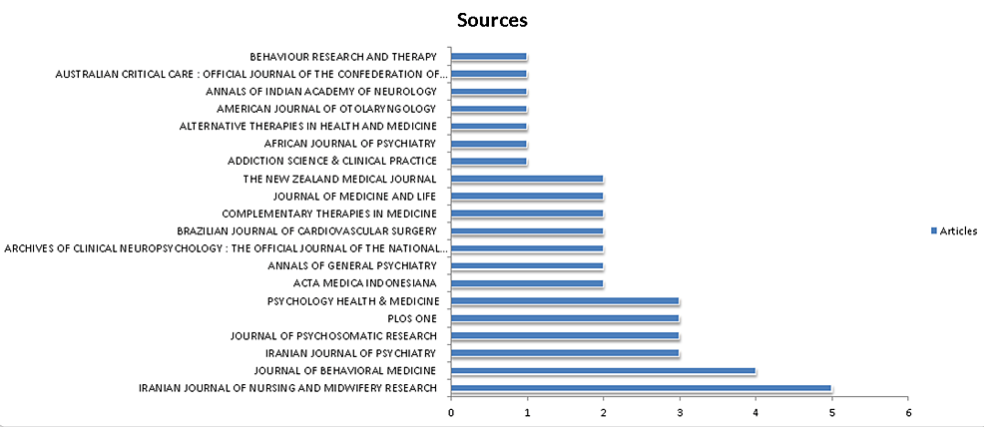
A graphical illustration of the most relevant journals with their article frequency.

**Figure 5:**
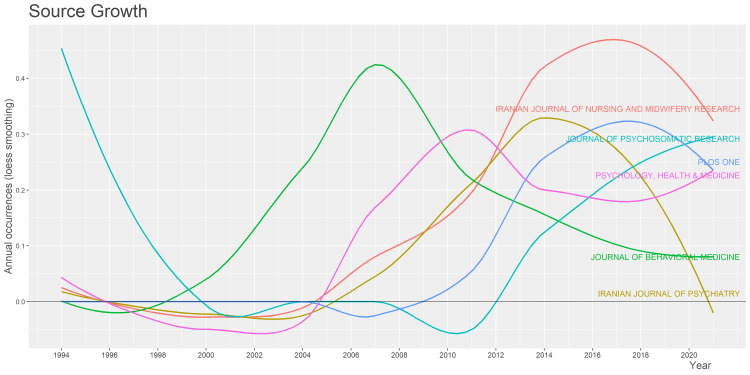
A graphical illustration of the Journals growth trend from the year 1994 to 2020.

The top journal as shown in Figure 4 in terms of the number of publications is the Iranian journal of nursing and mid-wifey research with five publications followed by the Journal of behavioral medicine with four publications. Iranian journal of psychiatry, Journal of Psychosomatic Research, Plos One, and Psychology Health and Medicine have three articles. Rest 14 journals have an article count of less than three. In Figure 5, the Iranian journal of nursing and mid-wifey represents the highest peak annual occurrence in the year 2017 with less smooth decline afterward. Journal of Behavioral Medicine, in the year 2007, has the peak annual occurrence.

Analysis of Words and Growth Trends

**Figure 6:**
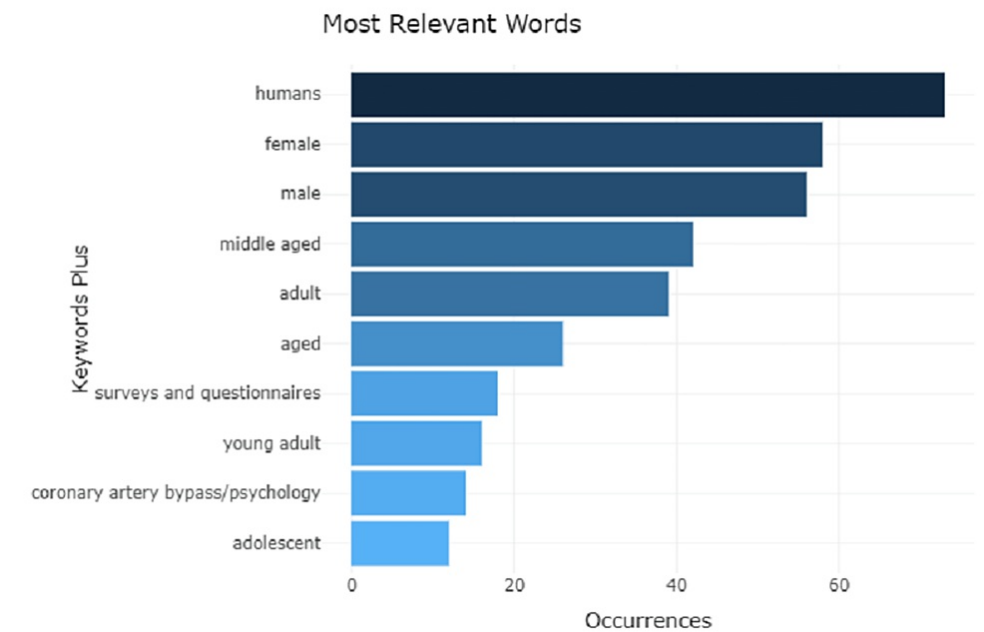
A graphical illustration of the occurrence of the most relevant words.

**Figure 7:**
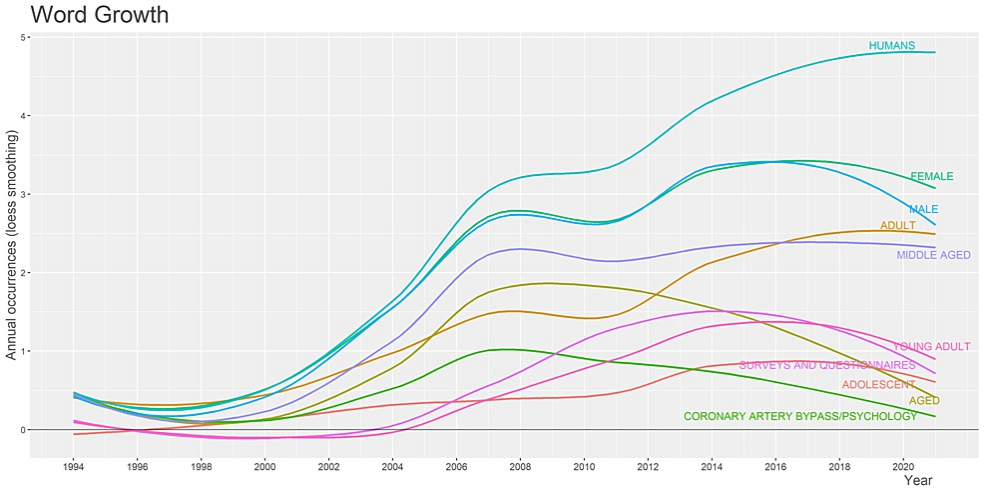
A graphical illustration of the word growth analysis.

The analysis of words (Figure 6) reveals that the most frequently used word is human (73) followed by female and male with frequencies 58 and 56, respectively, showing similar incidence among both genders. The word middle-aged with frequency represents the occurrence of CAD requiring CABG surgery is the highest, whereas the least occurrence words are the young adults and adolescents with frequency due to less incidence of CAD in this population. The growth rate in word frequency (Figure 7) shows an increasing trend over time. The word human remains at the peak followed by female and male shows a gradual rise from the year 2006 to 2020. There was no usage of the word young adults from the year 1996 to 2004.

Analysis of Global Scientific Production Trend

**Figure 8:**
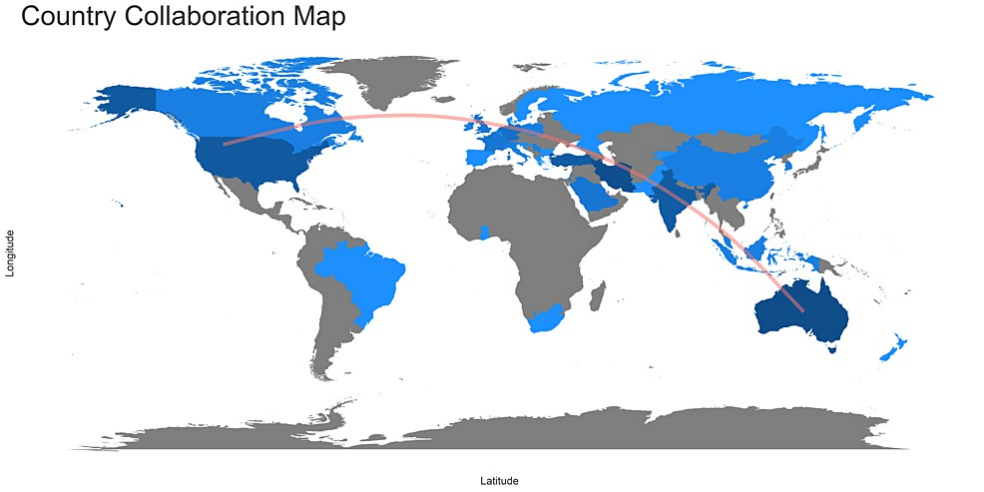
Global Map representing the collaboration of countries with the highest number of scientific publications.

**Figure 9:**
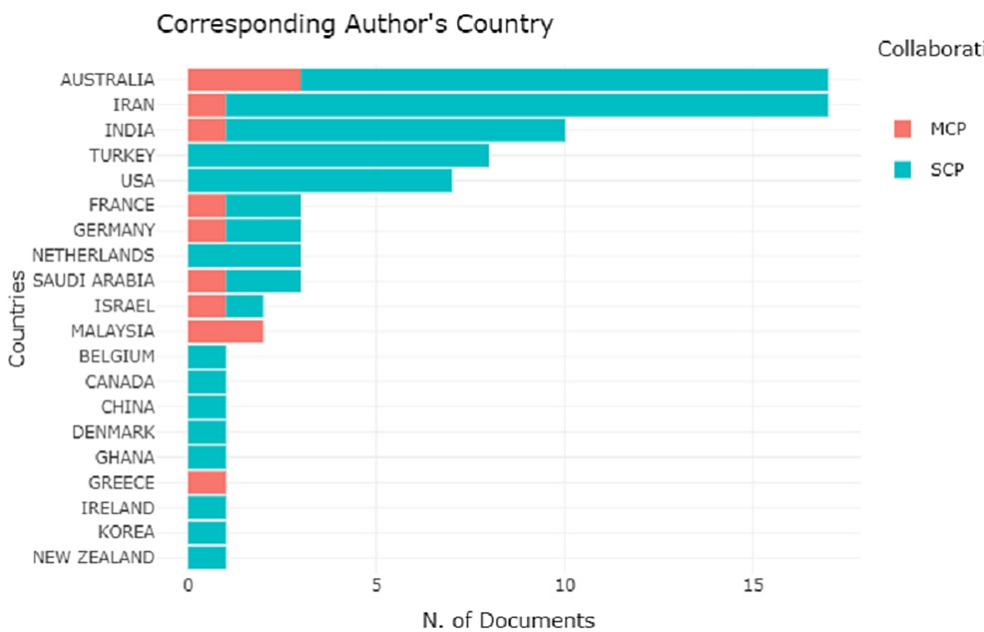
A graphical illustration of the analysis of corresponding authors’ countries with single country publications and multiple country publications.

The pictorial map in Figure 8 represents the collaboration of countries with the highest number of scientific publications. Evaluating the data based on country of origin, the majority of the identified scientific papers came from Australia, the United States of America, India, Iran, and Turkey. In Figure 9, an analysis of corresponding authors’ country with single country publication (SCP) and multiple country publication (MCP) reveals Australia and Iran leads with the highest scientific publication (17). Australia has 14 SCP and three MCP whereas Iran has 16 SCP and only one MCP. India, Turkey, and the USA follow the list with scientific publications 10, 8, and 7, respectively. Out of 20 countries, only 10 countries have multiple country publications with Australia at the peak with three MCP.

Analysis of University Affiliations

**Figure 10:**
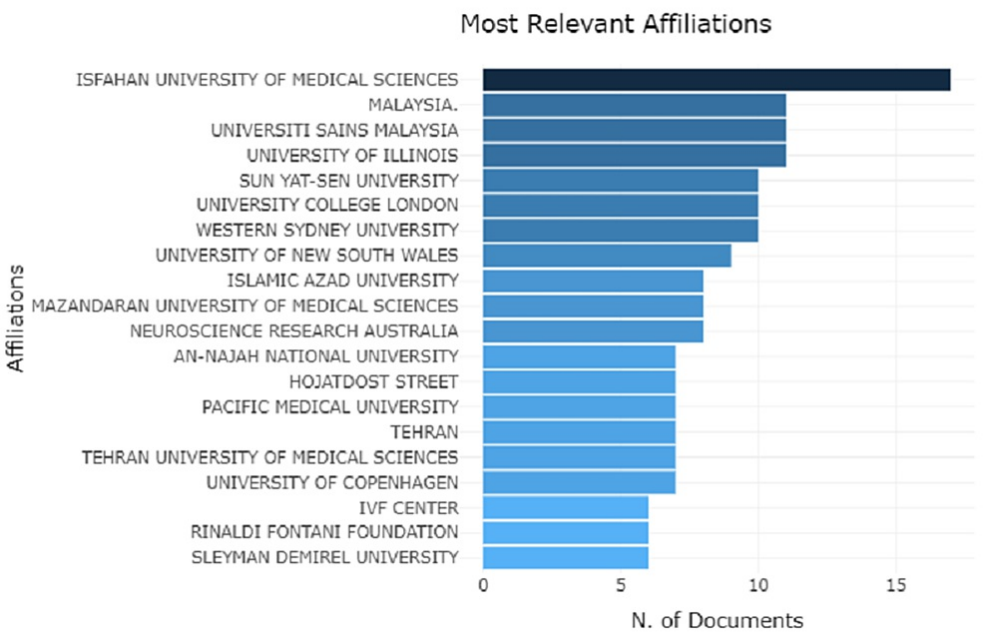
A graphical illustration of the analysis of university affiliations with total number of articles.

Examining the university affiliations reveals Isfahan University of Medical sciences leading the list with 17 articles. Malaysia, Universiti Sains Malaysia, and the University of Illinois follow the list with 11 articles. Whereas IVF center, Rinaldi Fontani foundation, and Sleyman Demirel University have the least contribution of six articles only (Figure 10).

Discussion

CABG is a traumatic procedure, and patients may experience worry, stress, anxiety, despair, and tension as a result. Psychological factors have an important role in the functional status and quality of life of patients who suffer CAD. Anxiety and depression symptoms are frequent, and they are linked to an increased risk of morbidity and mortality. The symptoms are more prevalent in patients who lack control and psychological benefits. The increasing prevalence of psychological issues in patients with cardiac surgery diverts the focus to promoting early identification, intervention, and psychological support to such patients [14]. Promoting psychological intervention anchored with cardiac rehabilitation might decrease the ill effect of depression on subsequent morbidity and mortality [15]. The interconnection between the cause of depression and its impact on CABG is a potentially promising area of cardiac surgery research for better understanding processes of emotional cardio-pathogenesis [16,17].

To date, the bibliometric study of cardiac surgery has provided the reader with some fascinating insights into the specialty's past and contemporary evolution [18]. It is more necessary to examine how quantitative evaluation may be made easy, clear, and comprehensible in the current state of knowledge than it is to focus excessively on precision, correctness, or scholarly concepts of purity. This bibliometric analysis was carried out to give a snapshot of research done between the years 1994 and 2020. In the global analysis, Australia and Iran are topped with 17 publications including both SCP and MCP followed by the USA, India, and Turkey. This shows the prevalence of psychological consequences and CABG is not limited to a particular continent.

Furthermore, spotting core content and research trends in particular domain keywords plays a major role [19,20]. The present analysis states that keywords like humans, male and female are most frequently used. Our analyses of authors and organizational associations revealed that Isfahan University of Medical Sciences has topped the list of research on psychological factors linked with CABG. The study concludes with a general overview of how such patients should be managed from a psychiatrist's standpoint. In the long term, a collaborative strategy is likely to be beneficial to the patient and cost-effective [21]. Patients should be screened before and after surgery as part of their normal workup. In a crowded outpatient setting, many patients may be unable to articulate their symptoms. Patient education and awareness may be critical in such situations.

Despite the possible limitations in bibliometric studies, this sort of analysis has intrinsic value. It facilitates debate and provides an overview of significant scientific research published in recent decades, as well as justification of patterns, themes, and eras depicted in the literature. Besides the absence of the earliest publications, this article gives a helpful viewpoint for individuals actively contributing to the corpus of cardiac surgical literature either as a practitioner or trainees. The topic of whether or not knowledge of history is important in the development and education of a cardiac surgeon is still a question. However, this bibliometric analysis shows the path on which the field has advanced in recent years.

## Conclusions

This study provides a reference on which to base future research, by identifying the most frequently cited publications, co-cited journals, collaborations between countries/ institutions/authors, and topic trends. The current study emphasizes the need for psychological screening in the management of CABG patients. This will lay the path for organizing and prioritizing future research on vascular psychiatry and its early management. It is, although, impaired by certain limitations but does provide an intriguing summary of the major publications on a subject and, in this case, illustrates the way in which cardiac surgery has developed in recent decades.
